# Quercetin Improves Cardiomyocyte Vulnerability to Hypoxia by Regulating SIRT1/TMBIM6-Related Mitophagy and Endoplasmic Reticulum Stress

**DOI:** 10.1155/2021/5529913

**Published:** 2021-03-29

**Authors:** Xing Chang, Tian Zhang, Qingyan Meng, ShiyuanWang, Peizheng Yan, Xue Wang, Duosheng Luo, XiuTeng Zhou, Ruifeng Ji

**Affiliations:** ^1^State Key Laboratory of Dao-di Herbs, National Resource Center for Chinese Materia Medica, China Academy of Chinese Medical Sciences, Beijing 100700, China; ^2^Guang'anmen Hospital of Chinese Academy of Traditional Chinese Medicine, Beijing 100053, China; ^3^Shandong University of Traditional Chinese Medicine, Jinan 250355, China; ^4^School of Business, Macau University of Science and Technology, Taipa, Macau 999078, China; ^5^School of Traditional Chinese Medicine, Guangdong Pharmaceutical University, Guangzhou 510006, China; ^6^Institute of Chinese Medicine, Guangdong Pharmaceutical University, Guangzhou 510006, China

## Abstract

Cardiomyocyte apoptosis is an important pathological mechanism underlying cardiovascular diseases and is commonly caused by hypoxia. Moreover, hypoxic injury occurs not only in common cardiovascular diseases but also following various treatments of heart-related conditions. One of the major mechanisms underlying hypoxic injury is oxidative stress. Quercetin has been shown to exert antioxidant stress and vascular protective effects, making it a promising candidate for treating cardiovascular diseases. Therefore, we examined the protective effect of quercetin on human cardiomyocytes subjected to hypoxia-induced oxidative stress damage and its underlying mechanism. Human cardiomyocytes were subjected to hypoxia/reoxygenation (H/R) in vitro with or without quercetin pretreatment; thereafter, flow cytometry, Cell Counting Kit-8 assay, laser scanning confocal microscopy, quantitative PCR, western blotting, and enzyme-linked immunosorbent assay were performed to analyze the effects of quercetin on cardiomyocytes. We found that H/R induced reactive oxygen species overproduction and endoplasmic reticulum stress, as well as inhibited the function of the mitochondria/endoplasmic reticulum and mitophagy, eventually leading to apoptosis and decreasing the viability of human cardiomyocytes. Quercetin pretreatment inhibited H/R-mediated overproduction of reactive oxygen species and damage caused by oxidative stress, increased mitophagy, regulated mRNA and protein expression of transmembrane BAX inhibitor-1 motif-containing 6 (TMBIM6), regulated endoplasmic reticulum stress, and improved the vulnerability of human cardiomyocytes to H/R. Furthermore, transfection with short interfering RNA against silent information regulator protein 1 (*SIRT1*) counteracted the protective effects of quercetin on cardiomyocytes. Thus, quercetin was predicted to regulate mitophagy and endoplasmic reticulum stress through SIRT1/TMBIM6 and inhibit H/R-induced oxidative stress damage. These findings may be useful for developing treatments for hypoxic injury-induced cardiovascular diseases and further highlight the potential of quercetin for regulating mitochondrial quality control and endoplasmic reticulum function.

## 1. Introduction

Myocardial hypoxia refers to abnormal changes in myocardial function induced in response to an insufficient O_2_ supply or metabolic disorders and is the primary cause of death [1, 2]. When cardiomyocytes cannot adapt to the lack of O_2_ supply, reactive oxygen species (ROS) are overproduced, leading to the development of mitochondrial quality control disorders and cardiomyocyte apoptosis. Hypoxia can also lead to dysfunctions in mitochondrial oxidative phosphorylation [3]. Moreover, excessive production of mitochondrial ROS because of mitochondrial respiratory chain dysfunction can affect signal transduction pathways, resulting in lipid peroxidation of the cell membrane and an imbalance in G protein and effector coupling [4]. Mitochondrial dysfunction and disruptions in homeostasis of the intracellular environment are accompanied by endoplasmic reticulum (ER) stress, which further reduces cell viability and accelerates apoptosis [5].

Quercetin (Que) is found in the flowers, leaves, and fruits of many plants, mostly in the form of glycosides [6]. As a natural antioxidant, Que exerts pharmacological effects such as regulating mitochondrial quality control and ER function, reducing capillary fragility, lowering blood lipids, and increasing coronary blood flow. Although Que has been demonstrated to protect cardiomyocytes [7], its underlying mechanism, particularly in the mitochondria and ER, is unclear.

Mitophagy is one of the most important pathways by which mitochondria maintain homeostasis [8], and a two-way regulatory mechanism is associated with hypoxia during cell metabolic stress [9]. Hypoxia activates mitophagy in various manners. Mitophagy can degrade damaged proteins to maintain cell survival [10]; however, when mitophagy is excessive, the cells are damaged and can undergo type II programmed cell death [11]. Studies have shown that mild hypoxia (1% or 0.1% O_2_) induces autophagy to promote cell survival but does not cause apoptosis [12]. Therefore, early stage hypoxia can induce protective autophagy and moderate apoptosis [13]. However, long-term exposure to hypoxia or extremely low O_2_ concentrations (0.1% or 0% O_2_) causes the death of numerous cells [14].

Hypoxia affects the function of multiple organelles, including the mitochondria and ER. In eukaryotic cells, the ER is the site of protein synthesis, modification, and folding [15]. Mitochondria are the most important organelles for supplying energy and regulating cell survival [16]. The ER and mitochondria share an interconnected region in which the molecular components are involved in an interaction mechanism to create a stable environment [17]. Homeostasis of this local environment enhances signal exchange between organelles and regulates the structure and function of mitochondria and the ER in the cardiovascular system [18].

Mitochondria interact with the ER structurally and functionally and are closely related to the survival of cardiomyocytes [17^,^^19^]. The adaptive response of the ER and mitophagy, as an intrinsic protective mechanism, promotes protein renewal and timely removal of harmful components, such as damaged proteins and mitochondria [20]. An imbalance in ROS-mediated redox signaling leads to the development of mitophagy disorder and ER stress, and the ability of the ER to fold proteins decreases, causing the accumulation of unfolded proteins and increased production of ROS [21]. These events eventually lead to apoptosis, necrosis, or inflammation [22].

Silent information regulator protein 1 (SIRT1) can regulate apoptosis, oxidative stress, mitochondrial constitution, and the ER [23]. Although Que can ameliorate myocardial ischemia-reperfusion-induced cardiomyocyte apoptosis via the SIRT1 pathway [24], the specific regulatory mechanism is unclear. TMBIM6 is an inhibitor of ER stress and was originally named as Bax inhibitor- (BI-) 12 [25]. TMBIM6, as a calcium channel-like protein, can reduce the calcium release balance on the ER membrane and plays an important role in regulating ER function and calcium homeostasis. TMBIM6 can play a protective role against various types of cell death caused by numerous stimuli [26]. Studies have shown that overexpression of TMBIM6 in cells can inhibit the activation of Bax, but its regulatory mechanism is unclear. As TMBIM6 and SIRT1 can regulate programmed cell death and autophagy, we predicted that TMBIM6 and SIRT1 are related in the regulation of mitophagy and ER stress.

Therefore, in this study, we examined the protective mechanism of Que against hypoxia/reoxygenation (H/R) stress in human cardiomyocytes and verified whether this antioxidant can improve the vulnerability of cardiomyocytes by regulating mitochondrial and ER functions through SIRT1/TMBIM6.

## 2. Materials and Methods

### 2.1. Cell Culture

Human cardiomyocytes were provided by the Chinese Academy of Medical Sciences (Institute of Basic Medicine, Beijing, China). Que (purity ≥ 98%) was provided by the Chinese Medicine Resource Center of the Chinese Academy of Traditional Chinese Medicine (Beijing, China). The cells were cultured in Dulbecco's modified Eagle medium (Gibco, Grand Island, NY, USA) containing 10% fetal bovine serum (Gibco) and penicillin-streptomycin (1%, Gibco) at 37°C (95% humidity and 5% CO_2_). The medium was exchanged every 2 days.

Before use in experiments, human cardiomyocytes were divided into four groups: (1) control group cells cultured under the abovementioned conditions; (2) H/R group cells cultured in fresh hypoxia medium (mM): CaCl_2_ 1.8, HEPES 20, KCl 10, MgSO_4_ 1.2, NaCl 98.5, NaHCO_3_ 6, NaH_2_PO_4_ 0.9, and sodium lactate 40 at 37°C; placed in an anoxic chamber for 3 h to induce hypoxia; then cultured in reoxygenation medium (mM): CaCl_2_ 1.8, glucose 5.5, HEPES 20, KCl 5, MgSO_4_ 1.2, NaCl 129.5, NaHCO_3_ 20, and NaH_2_PO_4_ 0.9 at 37°C; and finally exposed to an atmosphere of 95% O_2_ and 5% CO_2_ for 2 h; (3) Que group cells were treated with Que (50, 100, 150, 200, and 250 mg/L) for 24 h before H/R treatment; and (4) Que+*SIRT1* short interfering RNA (siRNA) group cells transfected with *SIRT1* siRNA for 36 h and then treated with 150 mg/L Que for 24 h before H/R treatment.

### 2.2. Measurement of Cardiomyocyte Viability

Human cardiomyocytes were healthy and grown to a confluence of >90%. Cells were digested with trypsin (0.25%; Sigma, St. Louis, MO, USA) for 10 min. Trypsin activity was terminated by adding Dulbecco's modified Eagle's medium, and cell counting was performed. The cells were then seeded into 12-well plates (50,000 cells/well), cultured for 12 h, and observed under an inverted microscope. Cell viability was measured by the Cell Counting Kit-8 (CCK-8) method.

### 2.3. Evaluation of Apoptosis

The different groups of cardiomyocytes were washed with phosphate-buffered saline. Using an apoptosis detection kit, the percentage of nonapoptotic cells was determined after biopsy staining. Cytomics FC 500 with CXP software (Beckman, Brea, CA, USA) was used for flow cytometric analysis.

### 2.4. Measurement of ROS Levels

Cells from different groups were collected and incubated with 2′,7′-dichlorofluorescin (10 *μ*M) at 37°C (20 min). ROS production was measured by flow cytometry.

### 2.5. Measurement of Mitochondrial Membrane Potential (MMP)

The MMP was measured in human cardiomyocytes by staining with JC-1 in the dark for 30 min. Images were acquired using a confocal microscope (Olympus, Tokyo, Japan).

### 2.6. Measurement of Antioxidant Enzyme Activity in Mitochondria

The activities of superoxide dismutase (SOD), glutathione (GSH), glutathione peroxidase (GPX), catalase (CAT), malondialdehyde (MDA), interleukin-10, interleukin-18, and tumor necrosis factor-*α* were determined using a total analysis kit.

### 2.7. Measurement of Mitochondrial Energy Metabolism

The mitochondrial O_2_ consumption rate was analyzed in real time in intact cells using an XFp Extracellular Flux Analyzer (Agilent Technologies, Santa Clara, CA, USA). Human cardiomyocytes were seeded at a density of 5 × 10^5^ cells/well, and mitochondrial energy metabolism was measured.

### 2.8. Western Blotting

Total protein was extracted from the cells in each group and resolved by electrophoresis (SDS-PAGE). The resolved peptides were electrotransferred onto polyvinylidene fluoride membranes and probed with primary antibodies against SIRT1, LC3A/B, Beclin-1, and Bcl-2 (Abcam, Cambridge, UK; diluted in 5% skim milk powder prepared in Tris-buffered saline) and TMBIM6 (Thermo Fisher Scientific Waltham, MA, USA; diluted in 5% skim milk powder prepared in Tris-buffered saline) at room temperature for 30 min and then at 4°C overnight. Goat anti-mouse IgG (H+L) conjugated with horseradish peroxidase (diluted 1 : 5000 in Tris-buffered saline) (Abcam, Cambridge, UK) served as the secondary antibody. The membrane was washed three times with Tris-buffered saline and incubated with electrochemiluminescence reagent for 3–5 min. An eBlot exposure instrument was used to expose the film for 60 s; images acquired after an appropriate exposure time were selected and analyzed using the ImageJ software (NIH, Bethesda, MD, USA).

### 2.9. Quantitative PCR

Total RNA extraction reagent (TriZol, Invitrogen, Carlsbad, CA, USA) was used to extract RNA from the cells. A NanoDrop® ND-2000 spectrometer (Wilmington, DE, USA) was used to determine the concentration and purity of RNA, and a PrimeScript™ RT reagent Kit with gDNA Eraser was used to reverse transcribe the RNA into cDNA. The cDNA was used as a template for real-time PCR, and the data were analyzed using the 2-△CT method [27].

### 2.10. Statistical Analysis

All experimental data are expressed as the mean ± standard error of the mean, and analysis of variance was used for overall evaluation. The *t*-test was used to analyze the differences between groups, and one-way or two-way analysis of variance and Tukey-test were used to analyze differences between multiple groups. *p* < 0.05 indicated a significant difference.

## 3. Results

### 3.1. Que Improves the Vulnerability of Human Cardiomyocytes to H/R

**Figure 1:**
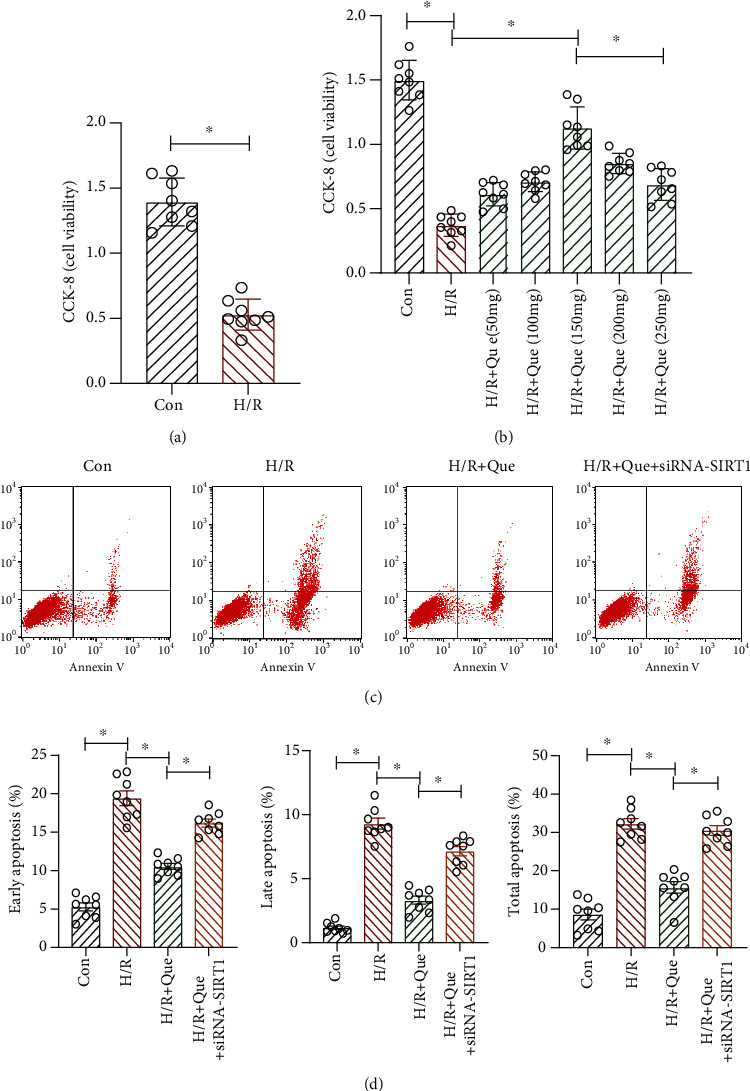
Quercetin (Que) ameliorates the vulnerability of human cardiomyocytes to hypoxia/reoxygenation (H/R): (a) viability of human cardiomyocytes; (b) viability of human cardiomyocytes with different concentrations of Que; (c, d) apoptosis of human cardiomyocytes before and after treatment. ^∗^*p* < 0.05. Con: control; *SIRT1* siRNA: *SIRT1* short interfering RNA.

Hypoxia was established by treating human cardiomyocytes with H/R, and the effect of Que on cell viability and apoptosis was evaluated. The CCK-8 assay showed that the viability of H/R-treated human cardiomyocytes was decreased significantly compared to that of the control group (Figure 1(a)). However, treatment of the cells with 50, 100, 150, 200, and 250 mg/L Que improved the viability of human cardiomyocytes after H/R treatment, with 150 mg/L Que inducing the most significant improvement (Figure 1(b)). Therefore, 150 mg/L Que was used in the apoptosis experiment. H/R increased the number of apoptotic human cardiomyocytes; 150 mg/L Que pretreatment significantly reversed this effect (Figures 1(c) and 1(d)). Moreover, transfection with *SIRT1* siRNA further reduced the viability of human cardiomyocytes and increased apoptosis (Figures 1(c) and 1(d)). These results indicate that H/R treatment promotes apoptosis and seriously reduces the viability of human cardiomyocytes, whereas Que can reverse these effects, ameliorating their vulnerability to H/R. Notably, the protective effect of Que was eliminated after SIRT1 knockdown, suggesting that SIRT1 mediates Que-induced protection of human cardiomyocytes.

### 3.2. Que Regulates Mitochondrial Homeostasis in H/R-Treated Human Cardiomyocytes through SIRT1

**Figure 2:**
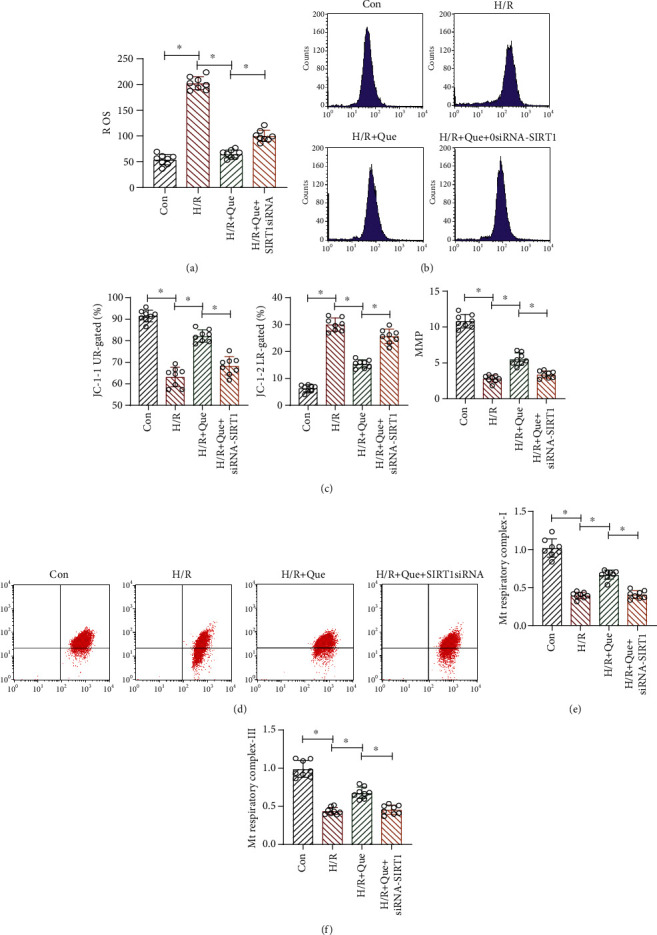
Quercetin (Que) regulates mitochondrial homeostasis in hypoxia/reoxygenation- (H/R-) stimulated human cardiomyocytes through SIRT1: (a, b) ROS level analysis; (c, d) MMP analysis; (e, f) activity of mitochondrial (Mt) respiratory complexes I/III. ^∗^*p* < 0.05. Con: control; *SIRT1* siRNA: *SIRT1* short interfering RNA.

Energy metabolism in cardiomyocytes mainly occurs in mitochondria, which can synthesize ATP through oxidative phosphorylation. As defects in mitochondrial energy metabolism and respiratory function may affect mitochondrial homeostasis, quality control of these organelles is critical. Therefore, we examined whether Que improved mitochondrial quality control through SIRT1 to ameliorate cardiomyocyte vulnerability to H/R. Cells treated with H/R exhibited increased mitochondrial ROS production compared to that in cells in the control group, a phenomenon that was inhibited upon pretreatment with Que (Figures 2(a) and 2(b)). Furthermore, H/R treatment reduced MMP levels, whereas pretreatment with Que reversed these effects (Figures 2(c) and 2(d)). H/R also inhibited the activity of mitochondrial respiratory complexes I/III (Figures 2(e) and 2(f)). Pretreatment with Que prevented these phenomena in a SIRT1-dependent manner, and SIRT1 knockdown eliminated the effect of Que on mitochondrial function (Figures 2(e) and 2(f)). Our results demonstrate that disruption of mitochondrial homeostasis triggered by H/R results in an imbalance in the levels of mitochondrial respiratory complexes, further disturbing mitochondrial homeostasis, decreasing human cardiomyocyte viability, and enhancing apoptosis. Thus, Que regulates mitochondrial homeostasis in human cardiomyocytes under H/R conditions through SIRT1.

### 3.3. Que Improves Mitochondrial Respiratory Function under Conditions of H/R through SIRT1

**Figure 3:**
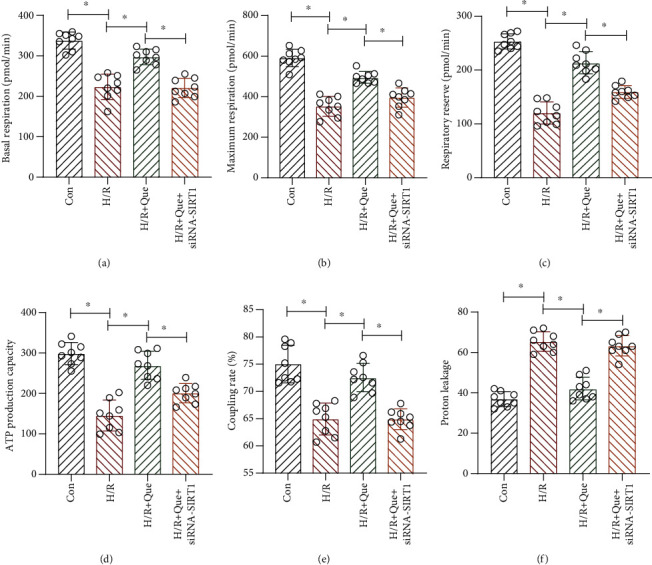
Quercetin (Que) ameliorates mitochondrial respiratory function under conditions of hypoxia/reoxygenation (H/R) through SIRT1: (a) basal mitochondrial respiration; (b) maximum respiration; (c) respiratory reserve; (d) ATP production capacity; (e) coupling rate; (f) proton leakage. ^∗^*p* < 0.05. Con: control; *SIRT1* siRNA: *SIRT1* short interfering RNA.

Mitochondria are extremely sensitive to changes in their environment, such as nutrition and O_2_ supplies, and respond through metabolic adaptations, eventually inducing an imbalance in mitochondrial homeostasis. When accompanied by abnormal respiratory chain function, mitochondrial respiratory function progressively declines, which culminates in ATP depletion, extensive cardiomyocyte damage, and apoptosis. To determine whether Que ameliorates mitochondrial respiratory function under conditions of H/R, we performed a mitochondrial stress test. The results revealed that compared to the control group, basal mitochondrial respiration (Figure 3(a)), maximum respiration (Figure 3(b)), respiratory reserve (Figure 3(c)), ATP production capacity (Figure 3(d)), and coupling rate (Figure 3(e)) were all significantly inhibited in human cardiomyocytes exposed to H/R. Moreover, proton leakage was significantly increased upon H/R exposure (Figure 3(f)). However, Que pretreatment reversed these effects (Figures 3(a)–3(f)). Interestingly, SIRT1 knockdown abrogated the effects of Que on mitochondrial respiratory function in H/R-exposed human cardiomyocytes (Figures 3(a)–3(f)). These data indicate that Que improves mitochondrial respiratory function and protects mitochondria in human cardiomyocytes through SIRT1. Mitochondrial respiration is central to energy production because it controls the coupling reaction of electron transfer in the respiratory chain complex in the inner mitochondrial membrane. ROS are by-products of electron transfer and the main cause of oxidative stress.

### 3.4. Que Ameliorates Mitochondrial Oxidative Stress Damage in H/R-Exposed Human Cardiomyocytes

**Figure 4:**
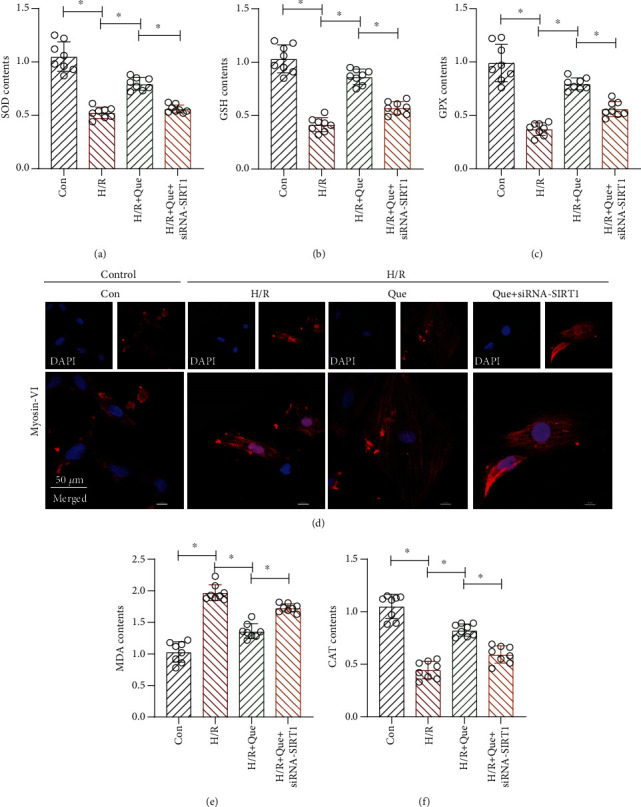
Quercetin (Que) ameliorates mitochondrial oxidative stress damage in hypoxia/reoxygenation- (H/R-) treated human cardiomyocytes: (a) antioxidant activity of superoxide dismutase (SOD), (b) glutathione (GSH), and (c) glutathione peroxidase (GPX); (d) myosin-VI detection by laser confocal microscopy; (e) activity of malondialdehyde (MDA); (f) antioxidant activity of catalase (CAT). ^∗^*p* < 0.05. Con: control; *SIRT1* siRNA: *SIRT1* short interfering RNA.

ROS-induced oxidative stress can induce the development of mitochondrial quality control disorders, which in turn lead to excessive mitochondrial lysis and mitophagy imbalance, inducing cardiomyocyte apoptosis. Next, we verified whether Que affects oxidative stress damage through SIRT1 under H/R by enzyme-linked immunosorbent assay. We found that Que reduced oxidative stress damage in human cardiomyocytes. Compared to the control group, the activities of SOD, CAT, GSH, and GPX in the H/R group decreased (Figures 4(a)–4(c) and 4(f)) and those of MDA increased (Figure 4(e)) in cells treated with H/R. Pretreatment of cells with Que inhibited these H/R-induced effects (Figures 4(a)–4(c), 4(e), and 4(f)); however, SIRT1 knockdown blocked Que-induced amelioration of oxidative stress damage (Figures 4(a)–4(c), 4(e), and 4(f)). Moreover, under hypoxia, myosin expression in cardiomyocytes decreased significantly after H/R, but Que reversed this phenomenon. The expression of myosin further decreased after transfection with si-*SIRT1* (Figure 4(d)). These results suggest that H/R inhibits the activity of the abovementioned antioxidant enzymes and causes oxidative stress damage and that Que regulates the mitochondrial redox state in hypoxic cardiomyocytes by activating SIRT1. Que may regulate myosin expression by maintaining mitochondrial/endoplasmic reticulum function, but further experiments are needed to verify this.

### 3.5. Que Regulates Human Cardiomyocyte Mitophagy under Conditions of H/R through SIRT1

**Figure 5:**
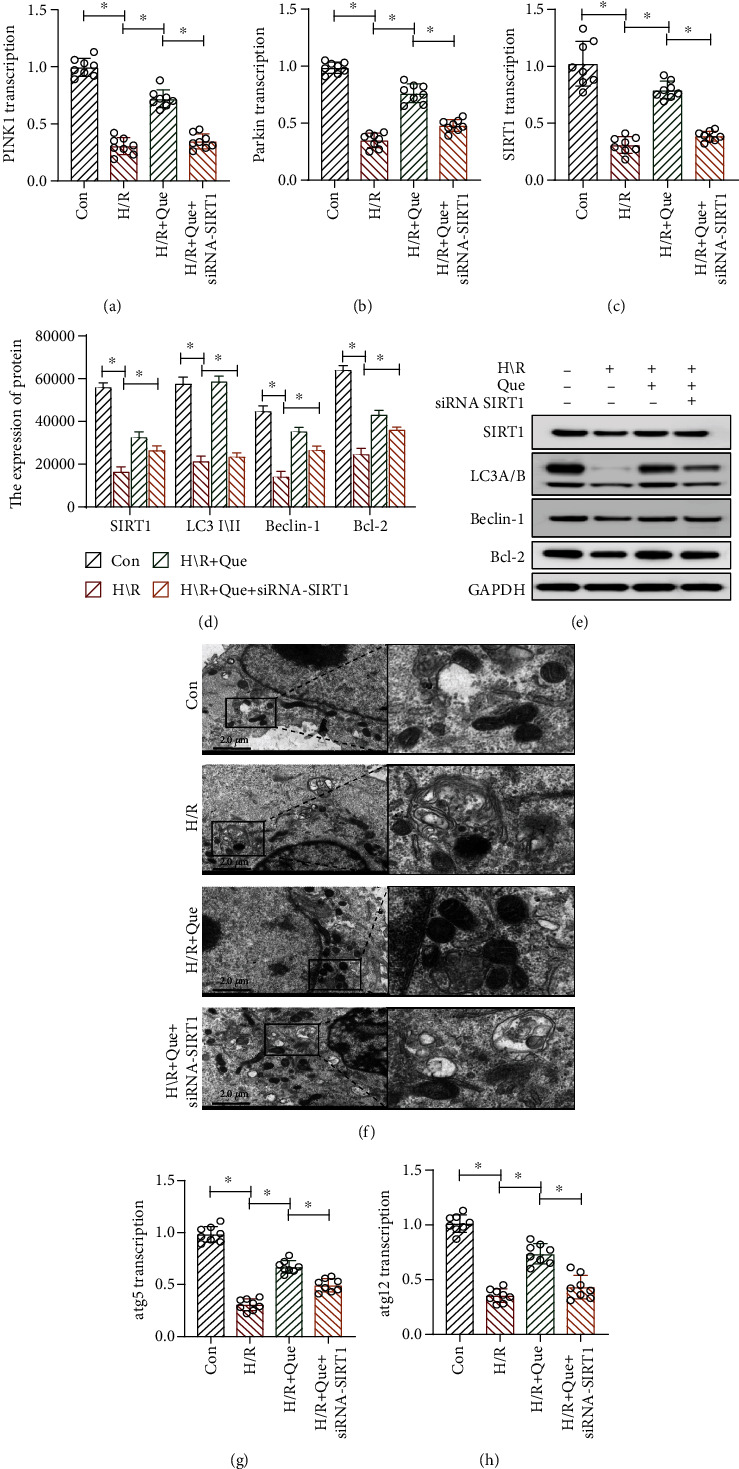
Quercetin (Que) regulates human cardiomyocyte mitophagy under conditions of hypoxia/reoxygenation (H/R): (a, b, c, g, h) levels of the *SIRT1*, *PINK1*/parkin, *ATG5*, and *ATG12* mRNAs determined by qPCR; (d, e) protein levels of SIRT1, LC3-I/II, Beclin-1, Bcl-2, and GAPDH determined by western blotting; (f) mitochondria observed by transmission electron microscopy. ^∗^*p* < 0.05. Con: control; *SIRT1* siRNA: *SIRT1* short interfering RNA.

Mitophagy is related to energy metabolism in human cardiomyocytes. Under physiological conditions, mitophagy can directly regulate mitochondrial numbers and ensure their normal function by clearing damaged and dysfunctional mitochondria from cells. These actions affect the physiological function of the mitochondrial respiratory chain and energy metabolism. In addition, mitophagy enables the removal of endogenous ROS produced by mitochondria. To explore the protective mechanism of Que in mitophagy, quantitative PCR was used to analyze the expression of *PINK1*/parkin and *ATG5/12*, which encode mitophagy-related proteins, and SIRT1. The results revealed that the expression of *PINK1*/parkin, *ATG5/12*, and *SIRT1* in the H/R group was lower than that in the control group (Figures 5(a)–5(c), 5(g), and 4(h)). However, under conditions of Que pretreatment, the expression of these mRNA was significantly increased (Figures 5(a)–5(c), 5(g), and 5(h)). Transmission electron microscopy revealed that the number of mitochondria in cells from the H/R group was lower than that in cells from the control group; Que pretreatment significantly increased this number (Figure 5(f)). Moreover, western blotting revealed that the levels of SIRT1, LC3-I/II, Beclin-1, and Bcl-2 were lower in the H/R group than in the control group, but Que reversed the expression of these proteins (Figures 5(d) and 5(e)). Notably, *SIRT1* knockdown eliminated the regulatory effect of Que on mitophagy (Figures 5(a)–5(h)).

### 3.6. Que Improves ER Stress in Human Cardiomyocytes under H/R Conditions through SIRT1/TMBIM6

**Figure 6:**
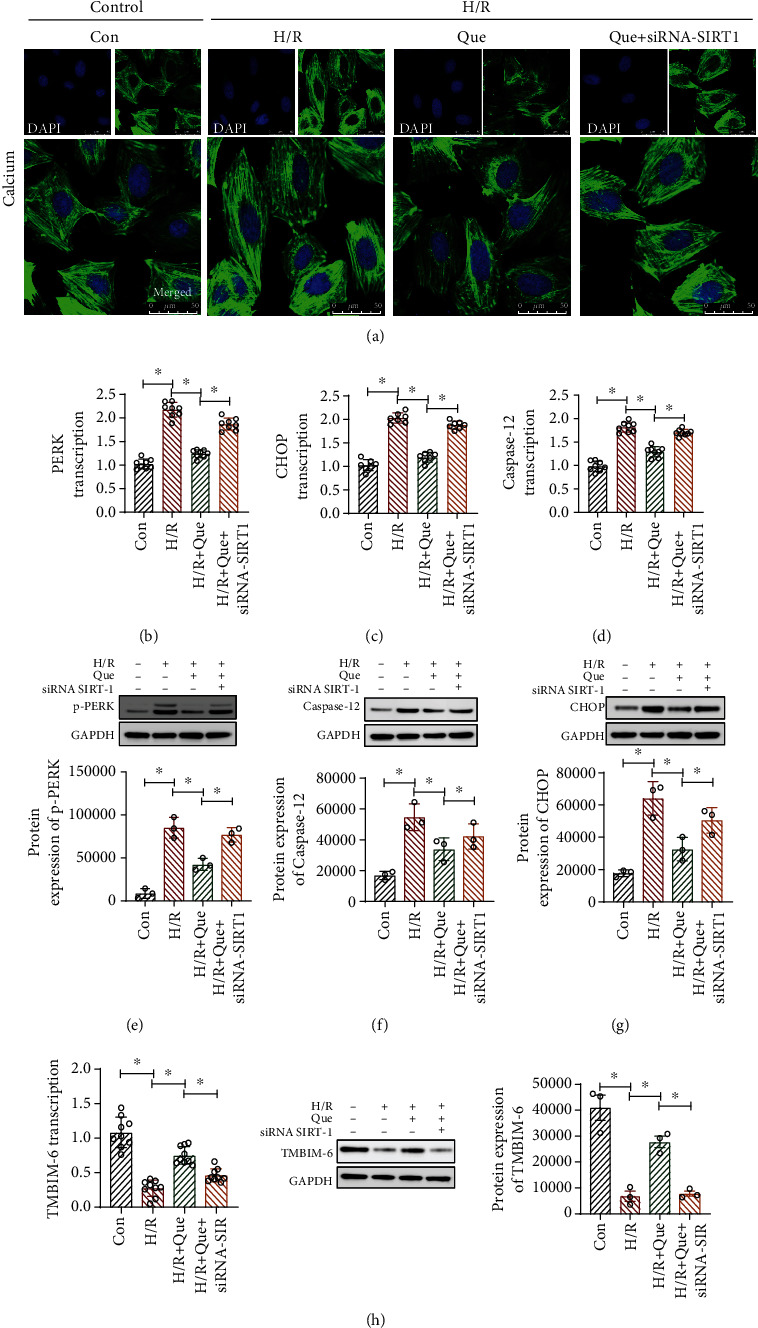
Quercetin (Que) ameliorates ER stress in human cardiomyocytes under conditions of hypoxia/reoxygenation (H/R): (a) calcium released from cardiomyocytes, as observed by laser confocal microscopy; (b–d) levels of the *PERK*, *CHOP*, and caspase-12 mRNAs determined by qPCR; (e–g) protein levels of p-PERK, CHOP, caspase-12, and GAPDH determined by western blotting; (h) mRNA and protein levels of TMBIM6 determined by qPCR and western blotting, respectively. ^∗^*p* < 0.05. Con: control; SIRT1 siRNA: SIRT1 short interfering RNA.

The above experimental results suggest that Que can enhance mitophagy and energy metabolism, inhibit oxidative stress damage, and ameliorate the vulnerability of cardiomyocytes to H/R. Additionally, the results demonstrate that SIRT1 plays a key role in Que-mediated regulation of mitochondrial function. To verify whether mitophagy inhibition under H/R conditions affects ER function and if Que-induced amelioration of cardiomyocyte vulnerability to H/R involves the regulation of ER function, we analyzed the mRNA levels of *PERK* and *CHOP*, which are closely related to ER stress, and caspase-12, which mediates ER stress-induced apoptosis. As shown in Figures 6(b)–6(d), compared to the control group levels, the mRNA and protein levels of PERK, CHOP, and caspase-12 were increased in human cardiomyocytes from the H/R group, whereas pretreatment of cells with Que inhibited this upregulation (Figures 6(b)–6(d)). Nevertheless, *SIRT1* knockdown countered the protective effect of Que (Figures 6(b)–6(d)). These results suggests that H/R upregulates ER stress-related proteins. As shown in Figure 6(a), H/R intervention can promote calcium release and lead to an imbalance in calcium homeostasis. However, Que inhibited calcium release from cardiomyocytes, and *SIRT1* siRNA abolished the ability of Que to regulate calcium homeostasis. TMBIM6 is an independent protein that can play a very important role in regulating ER function and Ca^2+^ homeostasis. TMBIM6 can interact with Ca^2+^ signaling proteins, inhibit apoptosis of the ER pathway, and regulate cell life and death. As shown in Figure 6(h), the mRNA and protein expression of TMBIM6, determined by qPCR and western blotting, respectively, decreased significantly under the influence of H/R. Pretreatment with Que reversed the effect of TMBIM6, and *SIRT1* siRNA inhibited the regulatory function of Que on TMBIM6. These results demonstrate that Que-mediated regulation of ER stress in human cardiomyocytes involves SIRT1/TMBIM6. However, the interaction mechanism between SIRT1 and TMBIM6 needs to be further clarified.

## 4. Discussion

We found that hypoxia increases ROS production and further induces oxidative stress damage, resulting in mitophagy dysfunction and ER stress and culminating in decreased myocardial cell viability. Que pretreatment can ameliorate mitophagy and mitochondrial function, regulate ER stress- and ROS-mediated oxidative stress damage, and restore myocardial cell viability. To understand the effect of Que on mitochondrial/ER function and to investigate whether its protective effect on cardiomyocytes involves the SIRT1 signaling pathway, we transfected cells with SIRT1 siRNA. Depletion of SIRT1 blocked the Que-induced regulation of mitochondrial/ER function and the protection of cardiomyocytes from H/R. Thus, we showed that Que regulates mitophagy through the SIRT1 signaling pathway and ameliorates the activity of cardiomyocytes under hypoxia conditions. Furthermore, H/R inhibited the mRNA and protein expression of TMBIM6. Que restored the expression of TMBIM6, whereas si*SIRT1* further inhibited it. Therefore, Que may regulate mitophagy and the ER through SIRT1/TMBIM6 to stabilize intracellular calcium homeostasis and improve cardiomyocyte activity.

Under physiological conditions in the vascular system, ROS levels can be increased through mitochondrial and nonmitochondrial pathways. Cells can then eliminate excessive ROS through the antioxidant system [28], resulting in reduced oxidative stress with respect to mitochondrial DNA, respiratory chain complex proteins, and other important molecules [29]. However, under hypoxic conditions, mitochondrial/ER dysfunction induces increased mitochondrial damage and excessive ROS production, resulting in MMP loss, along with the release of caspase and other apoptosis signaling molecules [30]. This is consistent with our findings showing that hypoxia-mediated oxidative stress damage increased ROS production, indirectly leading to mitochondrial/ER dysfunction and further damage in human cardiomyocytes.

Mitophagy is an autophagic process that selectively removes excess or damaged mitochondria [31]. Overexpression of ATG5, ATG12, and LC3-II (mitophagy-related proteins) significantly prolongs the lifespan of endothelial cells. These effects are closely related to increased MMP and ATP production, reduced mitochondrial DNA damage, and improved mitochondrial function [32]. Moreover, autophagy and mitophagy dysfunction may result in abnormal cardiac and endothelial cell function, including vascular aging and calcification [9]. Autophagy and selective mitophagy reverse endothelium-dependent diastolic dysfunction by regulating the total redox state of blood vessels, inhibiting ROS production, and increasing arterial stiffness [33].

These studies indicate that mitophagy plays a regulatory role in vascular and myocardial tissues and cells. A study of endothelial cells from elderly and young populations and aged and young mice found that ATG and Beclin-1 in the elderly groups were significantly decreased, suggesting decreased autophagy in aged vessels. Furthermore, autophagy inducers can reverse some changes in aging arteries and inhibit oxidative stress by increasing the bioavailability of nitric oxide [34]. In a mouse model of diabetic cardiomyopathy induced by a high-fat diet, parkin-mediated mitophagy reduced myocardial hypertrophy and diastolic dysfunction and protected cardiac function. In addition, pretreatment with Tat-Beclin-1 (a mitophagy inducer) reduced myocardial hypertrophy and diastolic dysfunction induced by continuous high-fat diet feeding. In this study, we found that Que upregulates Beclin-1 and LC3-I/II by regulating ATG, restoring the mitophagy level and energy metabolism function, and inhibiting ER stress and oxidative stress damage. These findings also corroborate the protective mechanism of mitophagy in cardiovascular diseases.

In addition to mitophagy dysfunction, ER dysfunction is an important factor leading to cardiomyocyte apoptosis [35]. Combined ER stress and mitophagy disorders lead to an imbalance in intracellular homeostasis [36]. When hypoxia-induced excessive ROS production mediates oxidative stress damage, unfolded proteins accumulate in the ER, exceeding the ability to perform the unfolded protein reaction, affecting mitophagy, and finally leading to increased myocardial apoptosis [37]. Que was found to inhibit ER stress, promote mitophagy, reduce mitochondrial dysfunction, and effectively maintain the homeostasis of myocardial cells by regulating SIRT1/TMBIM6 related to ER stress.

Previous research showed that Que regulates the SIRT1, JNK/c-JUN/CYP7A1, MAPK, and PI3K/AKT pathways [38]. Although this study preliminarily confirmed that Que can protect cardiomyocytes by regulating mitophagy and ER stress, its specific molecular mechanism is unclear. Another study showed that the axial shear stress of SIRT1-FOXO induces upregulation of SIRT1 and activates autophagy [39]. Moreover, SIRT1 can play a protective role in various cardiovascular diseases through diverse cellular functions. For instance, the SIRT1 activator SRT1720 can activate AMPK, enhance autophagy, and reduce myocardial apoptosis, whereas treatment with the SIRT1 inhibitor EX527 reverses the increase in autophagy. These findings suggest that SIRT1 promotes mitophagy, reduces hypoxia-induced apoptosis, and protects cardiomyocytes from oxidative stress. To further verify the regulatory pathway of the effects of Que on the ER through SIRT1, we examined the ability of Que to regulate TMBIM6.

As an important organelle, the ER can activate multiple ER stress signaling pathways to regulate protein folding, synthesis, protein degradation, gene expression, apoptosis, and bioenergy efficiency. Under various physiological and pathological conditions, unfolded proteins can accumulate in the ER, a cellular state known as ER stress. TMBIM6, as an important regulatory protein of ER stress, can participate in regulating ER stress signaling pathways. TMBIM6 can directly inhibit IRE1-*α* activity by binding to its cytoplasmic domain [40]. This protein is also responsible for controlling calcium release and the ER apoptosis pathway in the ER region [41]. Our results confirm that Que regulates mitophagy and ER stress through SIRT1/TMBIM6 and that the protective effect of Que on cardiomyocytes under oxidative stress damage caused by hypoxia is accomplished by SIRT-1/TMBIM6.

Nevertheless, this study had some limitations. First, although we inferred that Que regulates mitochondrial function and ER stress through SIRT1/TMBIM6 activation, the SIRT1–TMBIM6 interaction mechanism is not well-understood. Whether TMBIM6, as a regulatory protein of ER function, can directly regulate mitophagy is unknown. Further studies of the molecular mechanisms underlying these effects are needed to clarify the upstream and downstream processes regulating SIRT1/TMBIM6 expression. Second, the mechanism by which Que inhibits oxidative stress-induced damage in cardiomyocytes was only discussed in the context of mitophagy and ER stress; phenomena such as mitochondria–ER contact and calcium signal transduction were not explored. Therefore, in-depth studies are needed to determine the potential involvement of mitochondria–ER contact disorders in cardiomyocyte or vascular endothelial cell apoptosis. Finally, the therapeutic relevance of our in vitro findings must be verified in vivo and in clinical trials.

## 5. Conclusions

Our research shows that Que ameliorates the vulnerability of cardiomyocytes to H/R by inhibiting ROS production and oxidative stress damage, thereby improving mitophagy and energy metabolism, regulating mitochondrial/ER function, and ultimately reducing apoptosis. Under Que pretreatment, SIRT-1 and TMBIM6, which are closely related to the functions of mitochondria and ER, both showed significant improvement. The protective effect of Que on cardiomyocytes can be eliminated by depleting SIRT1, suggesting that Que regulates mitophagy and ER stress by activating SIRT1/TMBIM6 and inhibiting oxidative stress damage. These findings provide insights into the treatment of hypoxic injury-induced cardiovascular diseases and the potential of natural antioxidants for regulating mitochondrial and ER function.

## Data Availability

The data used to support the findings of this study are available from the corresponding author upon request.
